# Longitudinal Changes in Cholesterol Efflux Capacities in Patients With Coronary Artery Disease Undergoing Lifestyle Modification Therapy

**DOI:** 10.1161/JAHA.118.008681

**Published:** 2018-06-01

**Authors:** Marjorie Boyer, Valérie Lévesque, Paul Poirier, André Marette, Patricia L. Mitchell, Samia Mora, Patrick Mathieu, Jean‐Pierre Després, Éric Larose, Benoit J. Arsenault

**Affiliations:** ^1^ Centre de recherche de l'Institut universitaire de cardiologie et de pneumologie de Québec Canada; ^2^ Department of Medicine Faculty of Medicine Université Laval Québec Canada; ^3^ Department of Kinesiology Faculty of Medicine Université Laval Québec Canada; ^4^ Faculty of Pharmacy Université Laval Québec Canada; ^5^ Center for Lipid Metabolomics Divisions of Preventive and Cardiovascular Medicine Brigham and Women's Hospital Harvard Medical School Boston MA; ^6^ Department of Surgery Faculty of Medicine Université Laval Québec Canada

**Keywords:** adipose tissue, cholestrol efflux capacity, coronary artery disease, lifestyle, lipids and lipoproteins, Lifestyle, Risk Factors, Cardiovascular Disease, Lipids and Cholesterol

## Abstract

**Background:**

Our objective was to identify the determinants of high‐density lipoprotein cholesterol efflux capacity (HDL‐CEC) changes in patients with coronary artery disease who participated in a lifestyle modification program aimed at increasing physical activity levels and improving diet quality.

**Methods and Results:**

A total of 86 men with coronary artery disease aged between 35 and 80 years participated in a 1‐year lifestyle modification program that aimed to achieve a minimum of 150 minutes of aerobic physical activity weekly and improve diet quality. HDL‐CECs were measured before and after the 1‐year intervention using ^3^H‐cholesterol–labeled J774 and HepG2 cells. Visceral, subcutaneous, and cardiac adipose tissue levels were assessed before and after the intervention using magnetic resonance imaging. Lipoprotein particle size and concentrations were measured by proton nuclear magnetic resonance spectroscopy and a complete lipoprotein‐lipid profile was obtained. At baseline, the best correlate of HDL‐CECs were apolipoprotein AI (*R* ^2^=0.35, *P*<0.0001) and high‐density lipoprotein cholesterol (*R* ^2^=0.21, *P*<0.0001) for J774‐HDL‐CECs and HepG2‐HDL‐CECs, respectively. Baseline and longitudinal changes in HDL‐CECs were associated with several lipoprotein size and concentration indices, although high‐density lipoprotein cholesterol was the best predictor of longitudinal changes in J774‐HDL‐CECs (*R* ^2^=0.18, *P*=0.002) and apolipoprotein AI was found to be the best predictor of longitudinal changes in HepG2 cholesterol efflux capacities (*R* ^2^=0.21, *P*=0.002).

**Conclusions:**

Results of this study suggest that increases in high‐density lipoprotein cholesterol and apolipoprotein AI levels typically observed in patients with coronary artery disease undergoing healthy lifestyle modification therapy may be indicative of higher plasma concentrations of functional high‐density lipoprotein particles.

**Clinical Perspective**

**What Is New?**

- Our study identified the clinical and metabolic determinants of changes in high‐density lipoprotein cholesterol efflux capacity in patients with coronary artery disease undergoing lifestyle modification therapy.

**What Are the Clinical Implications?**

- Our observations suggest that changes in high‐density lipoprotein cholesterol and apolipoprotein AI levels typically observed in patients undergoing lifestyle modification therapy may be indicative of a higher circulating concentration of functional high‐density lipoprotein particles.

## Introduction

Over the past decades, prospective studies have documented the strong and consistent relationship between circulating high‐density lipoprotein (HDL) cholesterol levels and cardiovascular disease (CVD) risk.^1^ We have also recently shown that the inverse relationship between high levels of either HDL cholesterol (HDL‐C) or apolipoprotein AI (apo AI; the principal protein component of HDL particles [HDL‐Ps]) and CVD persists in patients who reach optimal levels of low‐density lipoprotein cholesterol with statin therapy.^2^ However, although the relationship between low HDL‐C levels and CVD is strong, whether this association reflects a causal link remains debated. Nevertheless, raising HDL‐C levels by pharmacotherapy has become an appealing strategy that has recently been tested for its potential to reduce cardiovascular events in high‐risk individuals. However, most if not all of these intervention studies have yielded disappointing results.^3^, ^4^, ^5^, ^6^, ^7^ This has led some to hypothesize that simply raising HDL‐C levels may not be sufficient, especially if HDL functional properties such as their ability to remove excess cholesterol from peripheral tissues, also known as cholesterol efflux capacities (CECs), are not concomitantly improved.

It is generally accepted that the most important role of HDL‐Ps is to promote reverse cholesterol transport, from resident lipid‐laden macrophages or foam cells in the atherosclerotic plaque back to the liver, which promotes cholesterol excretion via biliary salts in the feces.^8^ This process involves several membrane transporters such as ATP‐binding cassette (ABC) A1 and G1 as well as the scavenger receptor class B type 1 (SR‐B1), which interact mostly with apo AI. Recent studies have shown that HDL‐CEC may strongly predict CVD risk, even independently of HDL‐C levels.^9^, ^10^, ^11^, ^12^ Although the association between HDL‐CECs and cardiovascular outcomes is now established, whether HDL‐CEC represents a causal risk factor for CVD is unknown. Additionally, the determinants and correlates of HDL‐CECs are not well understood.

Several studies have shown that individuals with ectopic fat deposition, which refers to lipid accumulation in nonfat tissues such as the abdomen (visceral fat), liver, heart, muscle, and pancreas are at increased risk for CVD and type 2 diabetes mellitus.^13^, ^14^ Over the past years, studies have shown that individuals with a high accumulation of visceral and ectopic fat are not only characterized by lower HDL‐C, but that these HDL‐Ps are smaller and more dense.^15^ Few studies have sought to determine the potential association between visceral/ectopic fat accumulation on HDL function and CECs. Furthermore, although studies have shown that exercise and a healthy diet could increase HDL‐C and HDL‐P number, the potential impact of the related loss of ectopic fat on HDL‐CECs is also unknown.^16^, ^17^

The objectives of the present study were first to identify the clinical and biological factors associated with HDL‐CECs in patients with coronary artery disease, and second to determine whether these parameters could also predict the changes in HDL‐CECs observed in these patients after undergoing healthy lifestyle modification therapy.

## Material and Methods

The data, analytic methods, and study materials will not be made available to other researchers for purposes of reproducing the results or replicating the procedure.

### Study Participants and Study Design

A total of 86 men, aged between 39 and 79 years, were recruited at the Institut universitaire de cardiologie et de pneumologie de Québec (IUCPQ), after coronary artery bypass grafting surgery (range: from 25 to 516 days after surgery). All selected patients had severe coronary artery disease that required a coronary artery bypass grafting procedure according to current American College of Cardiology/American Heart Association practice guidelines.^18^ Exclusion criteria for the 1‐year lifestyle modification program included greater than moderate systolic dysfunction (left ventricular ejection fraction <40%), impaired renal function (creatinine>150 mmol/L), inflammatory or known autoimmune disease, and/or lung disease. Patients with significant valvular disease that may eventually require surgery were excluded, as were active smokers and patients taking weight‐loss medication. Each participant provided signed informed consent approved by the IUCPQ institutional review board. None of the patients had a cardiovascular event or a procedure during the 1‐year follow‐up. All study participants completed a 1‐year lifestyle modification program consisting of a personalized healthy eating strategy combined with physical activity counseling. Cardiometabolic risk variables were assessed at baseline and after the 1‐year lifestyle modification program. Physical activity counseling was performed by kinesiologists and nutritional follow‐up was realized by a registered dietitian. The frequency of visits was once a week for the first month, twice a month for the next 3 months, and once every 3 to 4 weeks for the rest of the intervention. The physical activity program aimed at achieving a minimum of 150 minutes of aerobic physical activity weekly at moderate to vigorous intensity (50%–80% of their maximum heart rate measured during maximal treadmill test). The personalized nutritional recommendations aimed to improve dietary quality in all participants and included moderate caloric restriction for overweight patients (−500 kcal daily). Briefly, patients were asked to increase daily consumption of fruits, vegetables, fibers, fish, and whole grains, as well as more proteins from vegetables, poultry, or fish. Patients were also advised to reduce saturated fatty acid intake and to decrease the consumption of food and beverages with added sugar and sodium.

### Body Composition Assessment

At baseline and follow‐up, each patient underwent anthropometric measurements with standardized procedures (weight, height, and waist circumference). In brief, waist circumference was obtained in the standard standing anatomical position and measurement was obtained at the end of normal expiration at the midpoint between the last rib and the iliac crest. Body mass index was calculated (weight in kg/height in m^2^). To measure adipose tissue (AT) volumes, magnetic resonance imaging was performed with a 1.5 Tesla Philips Achieva system during successive end‐expiratory breath holds (Philips Healthcare). In order to differentiate AT from other tissues, a 2‐dimensional gradient echo T1‐weighted sequence with and without fat saturation technique was performed. To evaluate abdominal adiposity, 5‐mm‐thick axial slices were performed at the level of the L4‐L5 intervertebral disc. Images were analyzed offline in a dedicated core laboratory by trained technicians blinded to patient data using semiautomated methodology previously described and reliability reported (QMass MR 7.0, Medis Medical Imaging Systems). Briefly, the signal intensity of AT on T1‐weighted imaging was defined by the identification of a section within the subcutaneous AT. The software determined the signal intensity range to identify AT by full‐width at half‐maximum method. Pixels in the intra‐abdominal space that corresponded to the signal intensity range previously defined by the software were considered as visceral AT. We also measured extracardiac AT volumes by analogous methodology, using dedicated cardiac coil during successive end‐expiratory breath holds. AT located between the myocardium and pericardial space was considered epicardial fat, while AT located on the outer surface of the pericardial space and within the mediastinum was considered pericardial fat.^19^, ^20^, ^21^ Image readers were blinded to patient characteristics, visit, and study hypothesis.

### Plasma Lipid‐Lipoprotein Profile, Laboratory Assays, and CECs

After a 12‐hour overnight fast, blood samples were collected from a forearm vein into Vacutainer tubes containing EDTA (Miles Pharmaceuticals) for the measurement of plasma lipid and lipoprotein levels. Plasma cholesterol and triglycerides concentrations were determined in plasma and lipoprotein fractions using a Technicon RA‐500 analyzer (Bayer Corporation).^22^, ^23^ The HDL fraction was obtained after precipitation of low‐density lipoprotein in the infranatant.^24^ Lipoprotein(a) levels were measured on the Cobas Integra Tina‐quant Lipoprotein(a) Gen 2 (Roche Diagnostics). Proprotein convertase subtilisin/kexin type 9 (PCSK9), adiponectin, leptin, tumor necrosis factor α and interleukin 6 levels were measured by ELISA. Lipoprotein particle concentrations and sizes were measured by proton nuclear magnetic resonance (NMR) spectroscopy at Laboratory Corporation of America Holdings (formerly LipoScience) using NMR LipoProfile analysis and LP3 deconvolution algorithm as previously described.^25^, ^26^, ^27^ GlycA levels were also quantified by NMR, and lipoprotein insulin resistance index scores were calculated as previously described.^28^, ^29^ Apolipoprotein B and apo AI levels were obtained in plasma and lipoprotein fractions by nephelometry using polyclonal antibodies on a Behring BN ProSpec (Dade Behring).^30^ High‐sensitivity C‐reactive protein (CRP) levels were measured by immunoassay (Dade Behring).^31^ As suggested by the Centers for Disease Control and Prevention/American Heart Association, individuals with high‐sensitivity CRP concentrations >10 mg/L were excluded.^32^ CECs of apolipoprotein B–depleted serum were measured using J774 macrophages and HepG2 hepatocellular carcinoma cells. Briefly, cells were plated and incubated in DMEM (J774) or EMEM (HepG2) media containing 1% bovine growth serum and 2 μCi of 3H‐cholesterol per milliliter for 24 hours. Cells were then equilibrated for 16 to 18 hours in DMEM (EMEM for HepG2 cells) containing 0.2% endotoxin‐free, low free fatty acids bovine serum albumin. Subsequently, cells were incubated with efflux medium containing 2.8% apolipoprotein B–depleted serum of study participants for 4 hours. Media were then collected and cells were harvested in NaOH 0.5 N. Liquid scintillation counting was used to measure the efflux of radiolabeled ^3^H‐cholesterol from the cells. CEC was calculated by the following formula: [Cpm in media/(Cpm in media+Cpm in cell lysates)]. Cholesterol efflux was also measured in serum‐free media, as previously described.^33^ The latter was subtracted from CECs using sera from the study participants. A control sample of plasma from healthy volunteers was also used in each plate. All assays were performed in triplicate and the preintervention and postintervention measurements of each study participant were measured on the same plate. Intra‐assay coefficients of variation for the efflux measurements were 8.0% for J774‐HDL‐CECs and 6.4% for HepG2‐HDL‐CECs.

### Cardiorespiratory Fitness and Hemodynamic Measurements

Cardiorespiratory fitness was assessed using a maximal treadmill test according to a modified Bruce protocol on a TMX 425 treadmill (Trackmaster) linked to a QuarkB2 monitor (Cosmed). The protocol began with a lower workload than the standard Bruce protocol at a speed of 1.7 mph at the first stage with 0% slope and with a speed of 1.7 mph with a 5% slope during the second stage.^34^ The third stage corresponded to the first stage of the standard Bruce protocol.^32^ Cardiorespiratory fitness (VO_2_ peak; mL/kg per minute) and heart rate at a standardized submaximal treadmill stage (1.7 mph, and 5% slope) were the 2 variables used in the assessment of fitness. The maximal treadmill test was conducted after coronary artery bypass grafting surgery and after the 1‐year intervention. Three blood pressure and pulse rate measurements were taken 3 minutes apart on the nondominant arm with an appropriate cuff size after the patient had been resting in the sitting position for 5 minutes before and after the intervention.

### Oral Glucose Tolerance Test

At baseline and at 1 year, after a 12‐hour overnight fast, a 75‐g oral glucose tolerance test was performed. Blood samples were taken at 0, 30, 45, 60, 90, 120, 150, and 180 minutes after the beginning of the test for the measurement of plasma glucose and insulin levels. The integrated areas under the curve of plasma glucose and insulin levels were calculated using the trapezoid method.^35^

### Statistical Analyses

The Shapiro‐Wilk procedure was used to test the normality of variables and variables with a nonnormal distribution were log‐transformed before statistical analysis. Univariable and multivariable linear regression models were used to determine which parameters significantly correlated with HDL‐CECs at baseline and during follow‐up. Spearman rank correlations were performed to determine the associations between HDL‐CECs and lipoprotein size and concentrations. Before each statistical test, HDL‐CECs values were divided by those of the control sample used in each plate, as previously described.^9^, ^11^ All statistical analyses were performed with SAS version 9.3 (SAS Institute).

## Results

Clinical characteristics and the cardiometabolic risk profile of the patients before the 1‐year lifestyle modification therapy are presented in Table 1. The mean age of the study participants was 62.3 (8.0) years. Of the 86 men included in this study, 17 had a body mass index >30 kg/m^2^, 21 had type 2 diabetes mellitus, 82 were treated with statins, and 11 were treated with ezetimibe. There were no patients taking niacin. At baseline, HDL‐CECs were comparable across different subgroups (for instance, in patients with versus without obesity and in patients with diabetes mellitus versus those without diabetes mellitus). HDL‐CECs were, however, lower in patients with low HDL‐C level (<1.03 mmol/L, data not shown).

**Table 1:** Baseline Characteristics of the Study Patients

No. of participants	86
Statin treatment	82 (95)
Body mass index, kg/m^2^	27.2±3.7
Waist circumference, cm	98.7±10.7
Abdominal AT, mL/5 mm	
Total	394.5±135.7
Subcutaneous	206.5±72.0
Visceral	189.7±89.6
Cardiac AT, mL/5 mm	30.6±12.0
VO_2_ peak, mL/min per kg	24.9±3.9
Plasma lipoproteins	
Total cholesterol, mmol/L	3.40±0.60
LDL‐C, mmol/L	1.60±0.42
HDL‐C, mmol/L	1.17±0.33
Triglycerides, mmol/L	1.39±0.73
apo B, g/L	0.67±0.10
apo AI, g/L	1.33±0.21
Fasting insulin, pmol/L	76.0±39.8
AUC insulin, pmol/L × 10^−3^	82.4±39.8
Lipoprotein(a), nmol/L	79.6±74.6
Leptin, ng/mL	6.93±4.39
Adiponectin, μg/mL	4.54±2.76
IL‐6, pg/mL	3.15±2.25
TNF‐α, pg/mL	2.37±1.18
PCSK9, ng/mL	268±69
CRP, mg/L	2.45±2.38

Values are indicated as mean±SD or number (percentage). apo indicates apolipoprotein; AT, adipose tissue; AUC, area under the curve; CRP, C‐reactive protein; HDL‐C, high‐density lipoprotein cholesterol; LDL‐C, low‐density lipoprotein cholesterol; TNF‐α, tumor necrosis factor α; PCSK9, proprotein convertase subtilisin/kexin type 9; VO_2_ peak, cardiorespiratory fitness.

To identify the cardiometabolic risk markers associated with the variance in HDL‐CECs, we performed multivariable linear regression analyses. Variables included in these analyses were those included in Table 1. As shown in Table 2, at baseline, apo AI and HDL‐C levels were strongly associated with the variance in J774‐HDL‐CECs. Cardiorespiratory fitness, visceral AT accumulation, and fasting insulin levels were also significantly associated with J774‐HDL‐CECs at baseline. In the multivariable model, HDL‐C and cardiorespiratory fitness levels explained 38.5% (*P*=0.05) of the variance in J774‐HDL‐CECs. HDL‐C and apo AI levels were also strongly associated with the variance in HepG2‐HDL‐CECs at baseline, with an additional minor contribution of visceral AT accumulation and fasting triglyceride and insulin levels. In the multivariable model, the variance in HepG2‐HDL‐CECs was explained by HDL‐C and triglycerides concentrations (*R* ^2^=26.8%, *P*=0.03). Although it was not found to be an independent predictor of HDL‐CECS, we found that CRP levels were associated with baseline J774‐HDL‐CECs (*r*=−0.24, *P*=0.03) and HepG2‐HDL‐CECs (*r*=−0.27, *P*=0.02). Other variables included in the model were not significantly associated with the variance in HDL‐CECs at baseline.

**Table 2:** Association Between J774‐HDL‐CECs and HepG2‐HDL‐CECs and Markers of the Cardiometabolic Risk Profile of Men at Baseline

Dependent Variables	Variables	Univariable Models		Multivariable Model		
		Beta (SE) (*P* Value)	*R* ^2^×100	Partial (*R* ^2^×100)	Total (*R* ^2^×100)	*P* Value
J774‐HDL‐CEC	apo AI	0.664 (0.10) (<0.0001)	35.1	35.1	35.1	<0.0001
	VO_2_ peak	0.302 (0.12) (0.01)	9.8	3.5	38.5	0.05
	HDL‐C	0.332 (0.06) (<0.0001)	28.9	···	···	···
	Visceral AT	−0.098 (0.04) (0.03)	8.9	···	···	···
	Insulin	−0.102 (0.04) (0.01)	8.7	···	···	···
HepG2‐HDL‐CEC	HDL‐C	0.302 (0.07) (<0.0001)	21.4	21.4	21.4	<0.0001
	Triglycerides	−0.012 (0.05) (0.79)	8.2	5.5	26.8	0.03
	apo AI	0.496 (0.12) (<0.0001)	18.9	···	···	···
	Insulin	−0.085 (0.04) (0.06)	4.7	···	···	···
	Visceral AT	−0.054 (0.05) (0.23)	4.3	···	···	···

apo indicates apolipoprotein; AT, adipose tissue; HDL‐C, high‐density lipoprotein cholesterol; HDL‐CECs, high‐density lipoprotein cholesterol efflux capacities; SE, standard error; VO_2_ peak, cardiorespiratory fitness.

**Figure 1:**
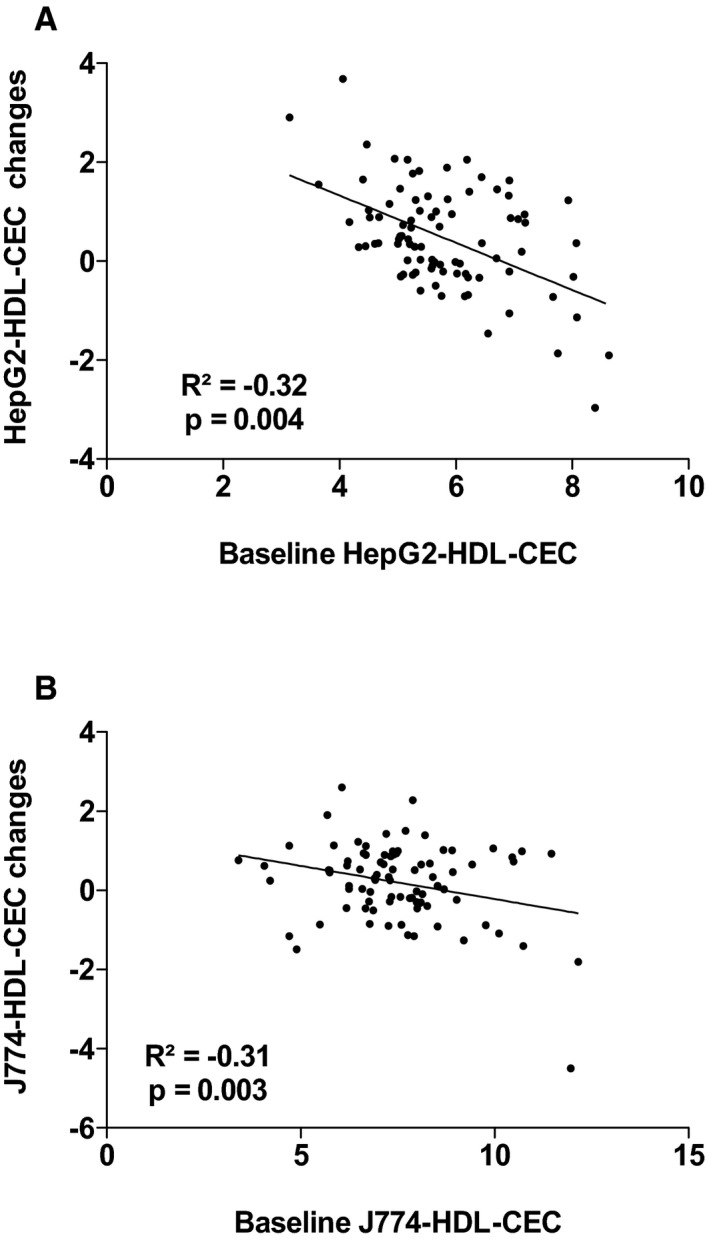
Association between HepG2–high‐density lipoprotein cholesterol efflux capacity (HepG2‐HDL‐CEC) changes and baseline HepG2‐HDL‐CEC (A) and J774‐HDL‐CEC changes with baseline J774‐HDL‐CEC (B).

Next, we investigated the association between the 1‐year changes in HDL‐CECs and changes in cardiometabolic risk markers. Results presented in Table 3 show that in the multivariable model, the variance in J774‐HDL‐CEC changes appeared to be solely explained by changes in HDL‐C (*R* ^2^=18.1%, *P*=0002). The other variables that contributed to the changes in the variance in J774‐HDL‐CEC were changes in apo AI, PCSK9, and triglyceride levels. Leptin and apo AI levels explained 38.9% (*P*=0.001) of the variance in HepG2‐HDL‐CECs in the multivariable model. The other variables that contributed to the changes in the variance in HepG2‐HDL‐CEC were changes in cardiac and subcutaneous AT accumulation and waist circumference. Figures 1 through 8 present the association between baseline HDL‐CECs and changes in HDL‐CECs, baseline HDL‐C and apo AI, and HDL‐CECs changes, baseline ectopic fat depots, and changes in HDL‐CECs as well as baseline VO_2_ peak and HDL‐CECs changes. These results suggest that changes in HDL‐CECs were negatively associated with baseline HDL‐CECs (with both cell types) and that changes in J774‐HDL‐CECs were associated with HDL‐C and apo AI changes (both not baseline HDL‐C or apo AI levels). On the other hand, changes in HepG2‐HDL‐CECs were associated with both baseline levels and changes in HDL‐C and apo AI. Interestingly, baseline HepG2‐HDL‐CEC was associated with baseline epicardial AT accumulation and changes in HepG2‐HDL‐CECs were associated with changes in epicardial AT accumulation.

**Table 3:** Association Between 1‐Year Changes in J774‐HDL‐CECs and HepG2‐HDL‐CECs and Changes in Markers of Cardiometabolic Risk Profiles

Dependent Variables	Variables	Univariable Models		Multivariable Model		
		Beta (SE) (*P* Value)	*R* ^2^×100	Partial (*R* ^2^×100)	Total (*R* ^2^×100)	*P* Value
J774‐HDL‐CEC	HDL‐C	0.137 (0.05) (0.009)	18.1	18.1	18.1	0.002
	apo AI	0.178 (0.07) (0.01)	5.99	···	···	···
	PCSK9	0.001 (0.001) (0.43)	4.85	···	···	···
	Triglycerides	0.025 (0.02) (0.30)	4.59	···	···	···
	Total cholesterol	0.054 (0.02) (0.01)	2.86	···	···	···
HepG2‐HDL‐CEC	apo AI	0.357 (0.08) (<0.0001)	21.2	21.2	21.2	0.002
	Leptin	−0.010 (0.01) (0.09)	18.1	17.8	38.9	0.001
	Cardiac AT	−0.004 (0.001) (0.02)	17.1	···	···	···
	Subcutaneous AT	−0.001 (0.001) (0.006)	15.3	···	···	···
	Waist circumference	−0.006 (0.001) (0.02)	14.6	···	···	···

apo indicates apolipoprotein; AT, adipose tissue; HDL‐C, high‐density lipoprotein cholesterol; HDL‐CECs, high‐density lipoprotein cholesterol efflux capacities; PCSK9, proprotein convertase subtilisin/kexin type 9; SE, standard error.

**Figure 2:**
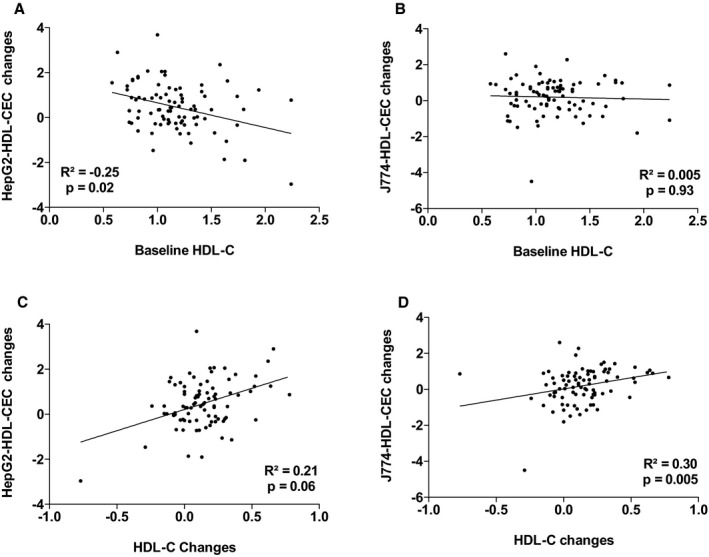
Association between HepG2–high‐density lipoprotein cholesterol efflux capacity (HepG2‐HDL‐CEC) changes and baseline high‐density lipoprotein cholesterol (HDL‐C; A) and HDL‐C changes (C) and J774‐HDL‐CEC changes with baseline HDL‐C (B) and HDL‐C changes (D).

**Figure 3:**
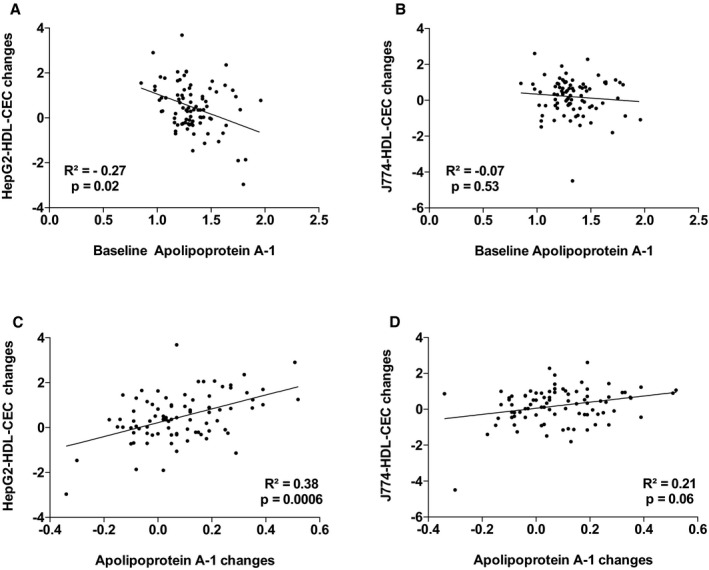
Association between HepG2–high‐density lipoprotein cholesterol efflux capacity (HepG2‐HDL‐CEC) changes and baseline apolipoprotein AI (apo AI; A) and apo AI changes (C) and J774‐HDL‐CEC changes with baseline apo AI (B) and apo AI changes (D).

**Figure 4:**
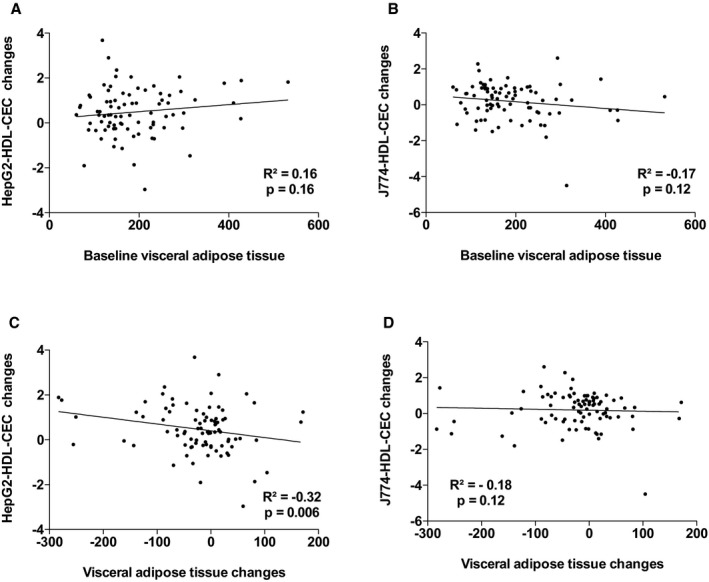
Association between HepG2–high‐density lipoprotein cholesterol efflux capacity (HepG2‐HDL‐CEC) changes and baseline visceral adipose tissue (AT; A) and visceral AT changes (C) and J774‐HDL‐CEC changes with baseline visceral AT (B) and visceral AT changes (D).

**Figure 5:**
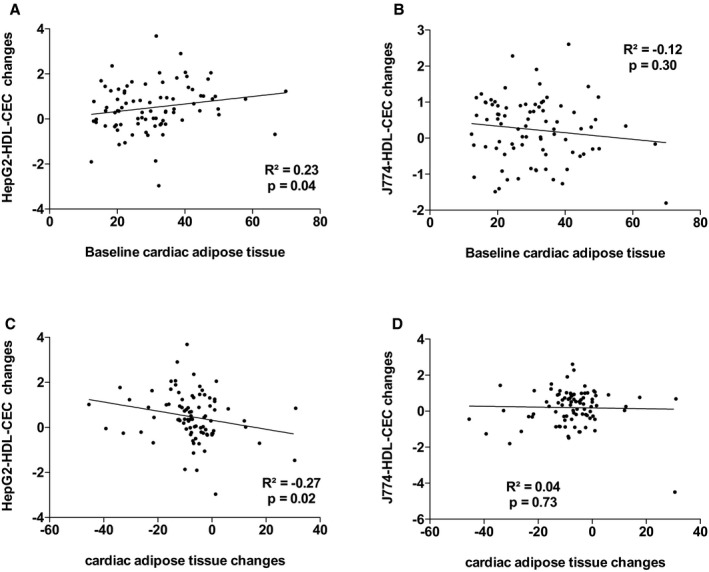
Association between HepG2–high‐density lipoprotein cholesterol efflux capacity (HepG2‐HDL‐CEC) changes and baseline cardiac adipose tissue (AT; A) and cardiac AT changes (C) and J774‐HDL‐CEC changes with baseline cardiac AT (B) and cardiac AT changes (D).

**Figure 6:**
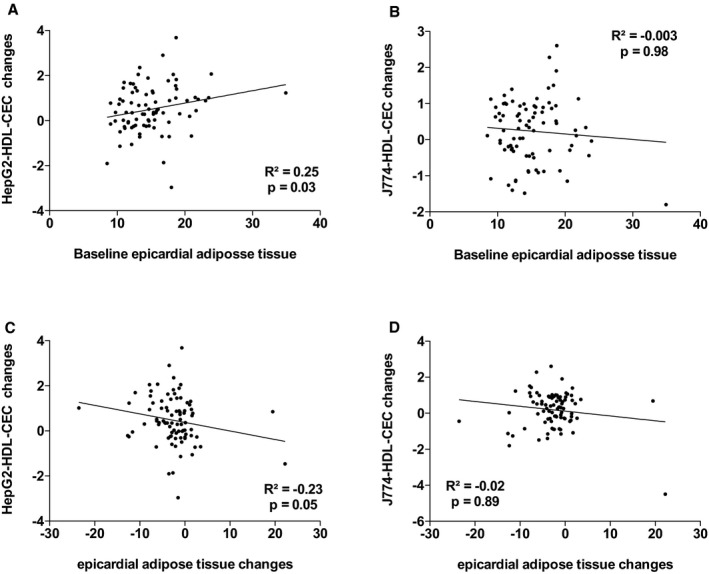
Association between HepG2–high‐density lipoprotein cholesterol efflux capacity (HepG2‐HDL‐CEC) changes and baseline epicardial adipose tissue (AT; A) and epicardial AT changes (C) and J774‐HDL‐CEC changes with baseline epicardial AT (B) and epicardial AT changes (D).

**Figure 7:**
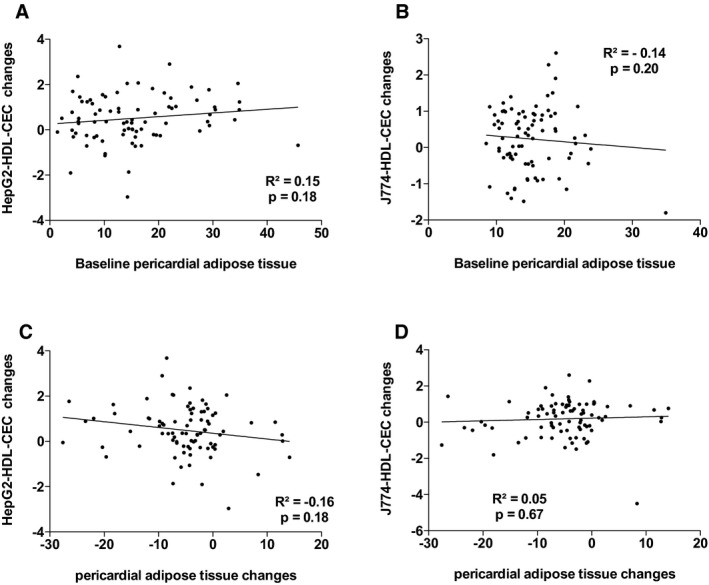
Association between HepG2–high‐density lipoprotein cholesterol efflux capacity (HepG2‐HDL‐CEC) changes and baseline pericardial adipose tissue (AT; A) and pericardia AT changes (C) and J774‐HDL‐CEC changes with baseline pericardia AT (B) and pericardia AT changes (D).

**Figure 8:**
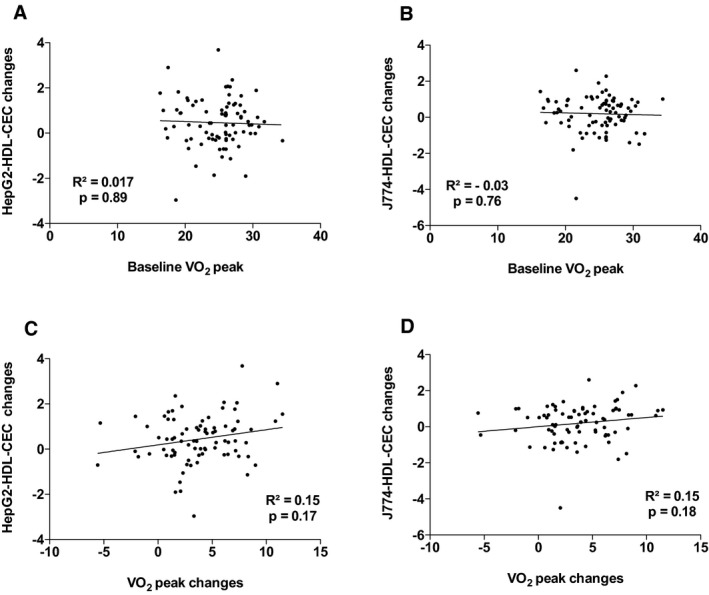
Association between HepG2–high‐density lipoprotein cholesterol efflux capacity (HepG2‐HDL‐CEC) changes and baseline cardiorespiratory fitness (VO_2_ peak; A) and VO_2_ peak changes (C) and J774‐HDL‐CEC changes with baseline VO_2_ peak (B) and VO_2_ peak changes (D).

We next sought to identify the correlates of HDL‐CECs measured by proton NMR spectroscopy. To avoid entering in the model variables that show evidence of colinearity, we assessed the correlation coefficients between changes in all NMR‐measured parameters and excluded 1 variable of each pair with a Spearman correlation coefficient (>0.50). We therefore excluded very low‐density lipoprotein and low‐density lipoprotein particle size. Table 4 shows the association between HDL‐CECs and NMR parameters at baseline. The best correlate of HDL‐CECs at baseline (J774‐ and HepG2‐HDL‐CECs) was the concentration of large HDL‐Ps. Table 5 shows the association between changes in HDL‐CECs and changes in these parameters. For both changes in J774‐ and HepG2‐HDL‐CECs, changes in the total number of HDL‐Ps appeared to be the best correlate of changes in HDL‐CECs, although the association appeared to be stronger for HepG2‐HDL‐CECs compared with J774‐HDL‐CECs.

**Table 4:** Association Between J774‐HDL‐CECs and HepG2‐HDL‐CECs and NMR Spectroscopy–Measured Lipoprotein Size and Concentration of Men at Baseline

Dependent Variables	Variables	Univariable Models		Multivariable Model		
		Beta (SE) (*P* Value)	*R* ^2^×100	Partial (*R* ^2^×100)	Total (*R* ^2^×100)	*P* Value
J774‐HDL‐CEC	Large HDL particles	0.166 (0.04) (<0.0001)	17.21	17.2	17.2	0.0004
	HDL particle size	0.105 (0.07) (0.13)	12.03	···	···	···
	Lp‐IR	−0.184 (0.06) (0.002)	10.85	···	···	···
	GlycA	−0.254 (0.08) (0.003)	8.44	···	···	···
	Small LDL particles	−0.111 (0.04) (0.004)	5.80	···	···	···
HepG2‐HDL‐CEC	Large HDL particles	0.132 (0.04) (0.003)	8.63	8.63	8.63	0.02
	HDL particle size	0.831 (0.45) (0.07)	3.25	···	···	···
	Lp‐IR	−0.128 (0.06) (0.04)	3.12	···	···	···
	Small LDL particles	−0.083 (0.04) (0.04)	2.88	···	···	···
	IDL particles	0.037 (0.03) (0.24)	2.15	···	···	···

HDL indicates high‐density lipoprotein; HDL‐CECs, high‐density lipoprotein cholesterol efflux capacities; IDL, intermediate‐density lipoprotein; LDL, low‐density lipoprotein; Lp‐IR, lipoprotein insulin resistance index; NMR, nuclear magnetic resonance; SE, standard error.

**Table 5:** Association Between 1‐Year Changes in J774‐HDL‐CECs and HepG2‐HDL‐CECs and Changes in NMR Spectroscopy–Measured Lipoprotein Size and Concentration

Dependent Variables	Variables	Univariable Models		Multivariable Model		
		Beta (SE) (*P* Value)	*R* ^2^×100	Partial (*R* ^2^×100)	Total (*R* ^2^×100)	*P* Value
J774‐HDL‐CEC	Total HDL particles	0.007 (0.002) (0.01)	6.71	6.71	6.71	0.02
	GlycA	−0.0002 (0.0001) (0.09)	3.34	···	···	···
	Large HDL particles	0.011 (0.007) (0.11)	2.99	···	···	···
	Small HDL particles	0.002 (0.002) (0.32)	1.16	···	···	···
	IDL particles	0.0001 (0.0002) (0.36)	0.98	···	···	···
HepG2‐HDL‐CEC	Total HDL particles	0.013 (0.003) (0.0002)	16.75	16.75	16.75	0.0002
	Large HDL particles	0.024 (0.007) (0.003)	11.15	···	···	···
	Small VLDL particles	−0.002 (0.001) (0.01)	7.49	6.21	22.96	0.01
	IDL particles	0.0003 (0.0002) (0.04)	5.24	···	···	···
	Medium VLDL particles	0.003 (0.001) (0.04)	5.10	···	···	···

HDL indicates high‐density lipoprotein; HDL‐CECs, high‐density lipoprotein cholesterol efflux capacities; IDL, intermediate‐density lipoprotein; NMR, nuclear magnetic resonance; SE, standard error; VLDL, very low‐density lipoprotein.

## Discussion

Over the past 5 years, results of Mendelian randomization studies as well as phase 3 randomized clinical trials testing the impact of HDL‐raising alleles or molecules on cardiovascular outcomes, respectively, have provided evidence that HDL‐C may be a marker rather than a causal risk factor for CVD. Typically, HDL‐C levels are lower in individuals who are abdominally obese, poorly fit, and insulin resistant compared with lean, active, or insulin‐sensitive individuals. Consequently, targeting HDL‐C levels without targeting the root causes of the low HDL‐C phenotype may not translate into clinical benefits in high‐risk patients. Also, recently, results of prospective epidemiological studies have suggested that irrespective of HDL‐C levels, HDL‐CECs may be strongly associated with cardiovascular outcomes. Yet, similar to HDL‐C, whether HDL‐CEC is a causal risk factor in the etiology of CVD is unknown. In contrast to HDL‐C, however, whether HDL‐CEC is a marker of an impaired cardiometabolic risk profile is unknown. In this study, we sought to evaluate the determinants of HDL‐CECs measured using 2 cell models (J774 macrophages and HepG2 hepatocytes) in men with coronary artery disease before and after undergoing lifestyle modification therapy. Our results indicate that in patients with documented coronary artery disease, among a large panel of cardiometabolic risk markers, the best predictors of HDL‐CECs and changes in HDL‐CECs following a 1‐year lifestyle modification were HDL‐C and apo AI levels. These observations suggest that changes in HDL‐C and apo AI levels typically observed in patients undergoing lifestyle modification therapy may be indicative of a higher circulating concentration of functional HDL‐Ps.

### Impact of Physical Activity and Diet on HDL Functionality

To the best of our knowledge, our study is the first to document the longitudinal changes in HDL‐CECs and their determinants in relation to lifestyle factors. Some studies have linked HDL‐CECs with cardiometabolic risk parameters and cardiovascular outcomes using J774‐radiolabeled cells with ^3^H‐cholesterol. Recently, a retrospective study by Koba et al^36^ showed that a 6‐month cardiac rehabilitation program increased HDL‐C levels and HDL‐CECs in a sample of 57 men and women following an acute coronary syndrome. As the cardiac rehabilitation program included statin intensification, smoking cessation, and diet and exercise counseling (all of which were associated with HDL function in other studies as well as in that study), it was not possible to determine the respective contribution of diet and exercise to the potential increases in HDL function. In that previous study, changes in HDL‐CECs did not show any associations with changes in exercise capacity. In the Dallas Heart Study, the cross‐sectional associations between HDL‐CECs (measured using fluorescent‐labeled cholesterol [BODIPY]) and cardiometabolic risk parameters were investigated in 2924 individuals with various ethnic backgrounds.^10^ There was also no association between HDL‐CECs and the self‐reported level of physical activity. HDL‐CECs did, however, appear to be higher among individuals who reported higher levels of alcohol consumption. A previous cross‐sectional study has shown that the mean plasma CECs from 25 endurance athletes was 16% higher than that of a group of 33 nonathletes.^37^ In that study, CECs were measured using RAW‐264.7 macrophages. Aicher et al^38^ reported that an intervention aimed at achieving weight loss through a reduction in caloric intake and increases in physical activity in 100 overweight or obese women (a study population considerably different than ours) was associated with reductions in HDL‐C levels. In that study, they also reported that ABCA1‐mediated CECs also decreased while ABCG1 and SR‐B1–mediated CECs remained unchanged. Interestingly, a recent study also showed that weight loss induced by Roux‐en‐Y bypass surgery induced significant increases of total plasma CECs via both SR‐B1 (58%) and ABCG1 (26%) pathways in severely obese women.^39^ Thus, differences in initial profiles of these groups of patients and types of cells and assays used may contribute to the discordant results reported so far regarding HDL‐CECs.

### Inflammation and HDL Functionality

Several lines of evidence suggest that HDL functionality may be impaired upon proinflammatory conditions. For instance, HDL‐CECs have been shown to be lower in patients with psoriasis compared with patients without psoriasis.^40^ Similar observations were reported in patients with rheumatoid arthritis and systemic lupus erythematosus.^41^ Additionally, in patients with rheumatoid arthritis, changes in CRP upon potent anti‐inflammatory RA treatments correlated with improved HDL‐CECs.^42^ In the JUPITER (Justification for the Use of Statins in Prevention: an Intervention Trial Evaluating Rosuvastatin) trial of patients with evidence of chronic inflammation but no prior CVD, baseline CEC was not associated with CVD, but CEC measured in patients after potent statin therapy were significantly inversely associated with CVD events.^12^ Patients with acute coronary syndrome (another proinflammatory condition) have also been shown to be characterized by impaired HDL‐CECs. Interestingly, the proteome of HDL might also undergo important changes during acute coronary syndrome, and increased serum amyloid A on HDL‐Ps isolated from patients with acute coronary syndrome were reported.^43^ Serum amyloid A is an acute phase reactant produced in large quantities during the acute phase response that has the potential to remodel the HDL protein cargo, thereby reducing its CECs. It was recently reported in patients with peripheral artery disease that supervised strength and treadmill exercise training were not associated with changes in HDL subfractions, CEC, or inflammatory markers. However, HDL structure and function (CEC) was inversely associated with interleukin 6 at baseline.^44^ In our study, both CRP levels and GlycA (a novel marker of inflammation and cardiovascular risk^45^) were associated with J774‐HDL‐CECs.

### HDL Functionality and HDL Physicochemical Properties

In our study, HDL‐CECs were significantly associated with HDL physicochemical properties measured by NMR spectroscopy such as total HDL concentration (HDL‐P). Although the Spearman correlation coefficient between HDL‐CECs and HDL‐Ps was similar to that of HDL‐CECs and HDL‐C measured by both cell types, our regression analysis kept HDL‐C but not HDL‐P in the variables linked with HDL‐CEC. The discrepancies observed between the predictors of changes in J774‐HDL‐CECs and HepG2‐HDL‐CECs could be explained by differences in HDL physicochemical properties such as size and density. Indeed, in a study that included 174 patients with and without aortic valve disease, we have shown that HDL‐P size (NMR spectroscopy) correlated with HepG2‐HDL‐CECs but less consistently with J774‐HDL‐CECs.^33^ Similar findings were reported by Asztalos et al^46^ who measured HDL‐P size by 2‐dimensional gel electrophoresis. In this regard, it has been shown by Mweva et al^47^ that cholesterol efflux from hepatic cells to HDL‐Ps is mostly mediated by the SR‐B1 transporter, whereas the efflux from J774 to HDL‐Ps is mostly mediated by the ABCG1 and ABCA1 transporters. Additionally, on top of particle size, the amount and type of lipids such as phospholipids may render HDL‐Ps more efficient in promoting cholesterol efflux via SR‐B1 or ABCA1/G1 transporters.^48^

### HDL Functionality and Cardiometabolic Risk

Several studies have shown that visceral AT is an important feature of an impaired cardiometabolic risk profile closely associated with HDL physicochemical properties such as smaller HDL‐Ps.^15^ For instance, investigators of the CODAM (Cohort on Diabetes and Atherosclerosis Maastrich) study recently reported that HDL‐CECs (measured using THP‐1 macrophages) were lower in patients with metabolic syndrome (who are frequently characterized by increased visceral AT and insulin resistance).^49^ In our study, visceral AT was negatively associated with baseline HDL‐CECs measured with both cell types. One of the mechanisms through which visceral AT mobilization could improve HDL‐CECs may be caused by the effect of weight loss on cholesteryl ester transfer protein. Indeed, AT is an important source of circulating cholesteryl ester transfer protein, and prolonged physical inactivity has been linked with increased cholesteryl ester transfer protein activity in humans.^50^, ^51^ Studies have also reported reductions in plasma cholesteryl ester transfer protein activity and increases in HDL‐P size following moderate weight loss in obese patients.^52^ In the EPIC‐Norfolk (Norfolk Cohort of the European Prospective Investigation of Cancer) study,^53^ we have previously shown that the number of cardiometabolic risk factors (waist circumference, systolic blood pressure, triglycerides, apolipoprotein B, CRP, HDL‐C, and low‐density lipoprotein particle size) was associated with lower mean HDL‐P size. In 2002, Kraus et al also showed that a high amount of exercise training performed at high intensity (the equivalent of running ≈32 km per week at 65% to 80% of VO_2_ peak) could improve HDL‐P size in overweight/obese sedentary men and women.^16^ Despite, in our study, body fat distribution measurements such as visceral AT explained a minimal proportion of the variation in HDL‐CECs. In our study, 17.8% of the variance in HepG2‐HDL‐CEC changes appeared to be explained by variation in leptin levels. A few studies have assessed the association between HDL‐C and leptin levels and despite heterogeneity of the study populations, no associations were found.^54^, ^55^ In addition, one study evaluated the association between leptin levels and HDL‐CEC in 47 individuals with metabolic syndrome and no significant associations were found.^56^ A study on leptin‐deficient mice showed that this adipokine could, however, be an important regulator of hepatic SR‐B1 expression and thus play a role in metabolism.^57^ Additionally, our results suggest that up to 80% of the variance in HDL‐CECs could not be explained by the variation in the comprehensive list of phenotypes that we have obtained, thereby suggesting that more work is needed to identify the determinants in HDL‐CECs.

## Conclusions

HDL‐CECs have been shown to be strongly associated with the incidence of CVD in epidemiological studies, and HDL‐C–raising compounds have failed to provide any cardiovascular benefits in large cardiovascular outcomes trials. These observations combined with results from our study suggest that simply raising HDL‐C and/or HDL‐CECs without improving the strong driver of cardiometabolic risk that is ectopic fat accumulation may not be sufficient to reduce CVD incidence in secondary prevention.

## Sources of Funding

This study was supported by a team grant (No. 161971) from the Canadian Institutes of Health Research (CIHR). The measurement of CECs was supported by a grant from the Banting Research Foundation, Canada. B.A. holds a junior scholar award from the Fonds de recherche du Québec: Santé (FRQS). P.P. holds a senior scholar award from the FRQS. P.M. is an FRQS Research Chair on the Pathobiology of Calcific Aortic Valve Disease. A.M. is a Pfizer/CIHR Research Chair on the Pathogenesis of Insulin Resistance and Cardiovascular Disease. É.L. holds a research scholar award from FRQS and the Research & Innovation Chair in Cardiovascular Imaging from Université Laval. S.M. received funding from National Institutes of Health grants HL117861, HL134811, and DK 112940.

## Disclosures

None.

## Supporting information


**Figure S1.** Association between HepG2–high‐density lipoprotein cholesterol efflux capacity (HepG2‐HDL‐CEC) changes and baseline HepG2‐HDL‐CEC (A) and J774‐HDL‐CEC changes with baseline J774‐HDL‐CEC (B).
**Figure S2.** Association between HepG2–high‐density lipoprotein cholesterol efflux capacity (HepG2‐HDL‐CEC) changes and baseline high‐density lipoprotein cholesterol (HDL‐C; A) and HDL‐C changes (C) and J774‐HDL‐CEC changes with baseline HDL‐C (B) and HDL‐C changes (D).
**Figure S3.** Association between HepG2–high‐density lipoprotein cholesterol efflux capacity (HepG2‐HDL‐CEC) changes and baseline apolipoprotein AI (apo AI; A) and apo AI changes (C) and J774‐HDL‐CEC changes with baseline apo AI (B) and apo AI changes (D).
**Figure S4.** Association between HepG2–high‐density lipoprotein cholesterol efflux capacity (HepG2‐HDL‐CEC) changes and baseline visceral adipose tissue (AT; A) and visceral AT changes (C) and J774‐HDL‐CEC changes with baseline visceral AT (B) and visceral AT changes (D).
**Figure S5.** Association between HepG2–high‐density lipoprotein cholesterol efflux capacity (HepG2‐HDL‐CEC) changes and baseline cardiac adipose tissue (AT; A) and cardiac AT changes (C) and J774‐HDL‐CEC changes with baseline cardiac AT (B) and cardiac AT changes (D).
**Figure S6.** Association between HepG2–high‐density lipoprotein cholesterol efflux capacity (HepG2‐HDL‐CEC) changes and baseline epicardial adipose tissue (AT; A) and epicardial AT changes (C) and J774‐HDL‐CEC changes with baseline epicardial AT (B) and epicardial AT changes (D).
**Figure S7.** Association between HepG2–high‐density lipoprotein cholesterol efflux capacity (HepG2‐HDL‐CEC) changes and baseline pericardial adipose tissue (AT; A) and pericardia AT changes (C) and J774‐HDL‐CEC changes with baseline pericardia AT (B) and pericardia AT changes (D).
**Figure S8.** Association between HepG2–high‐density lipoprotein cholesterol efflux capacity (HepG2‐HDL‐CEC) changes and baseline cardiorespiratory fitness (VO2 peak; A) and VO2 peak changes (C) and J774‐HDL‐CEC changes with baseline VO2 peak (B) and VO2 peak changes (D).
