# Supplementary material for: Longitudinal Changes in Cholesterol Efflux Capacities in Patients With Coronary Artery Disease Undergoing Lifestyle Modification Therapy
Source: J Am Heart Assoc. 2018 Jun 1;7(11):e008681. doi: 10.1161/JAHA.118.008681 (PMC6015361; doi:10.1161/JAHA.118.008681)

SUPPLEMENTAL MATERIAL

Figure S1. Association between HepG2-HDL-CEC changes and baseline HepG2-HDL-CEC (A) and J774-HDL-CEC changes with baseline J774-HDL-CEC (B).

A

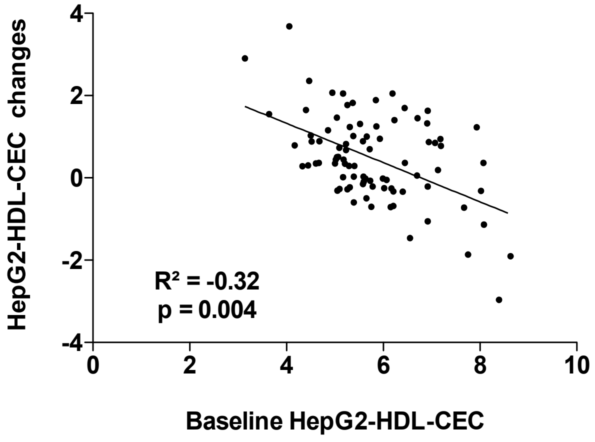

B

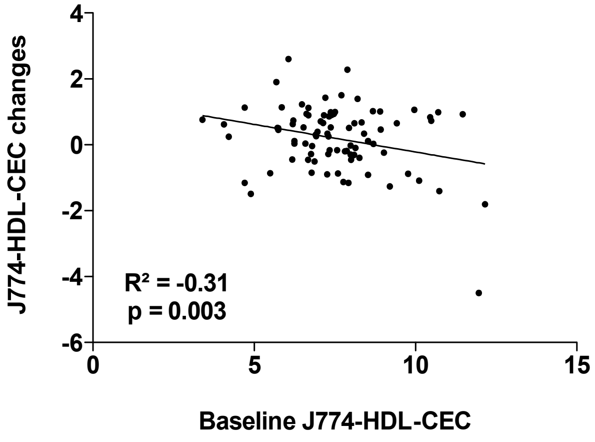

Figure S2. Association between HepG2-HDL-CEC changes and baseline HDL-C (A) and HDL-C changes (C) and J774-HDL-CEC changes with baseline HDL-C (B) and HDL-C changes (D).

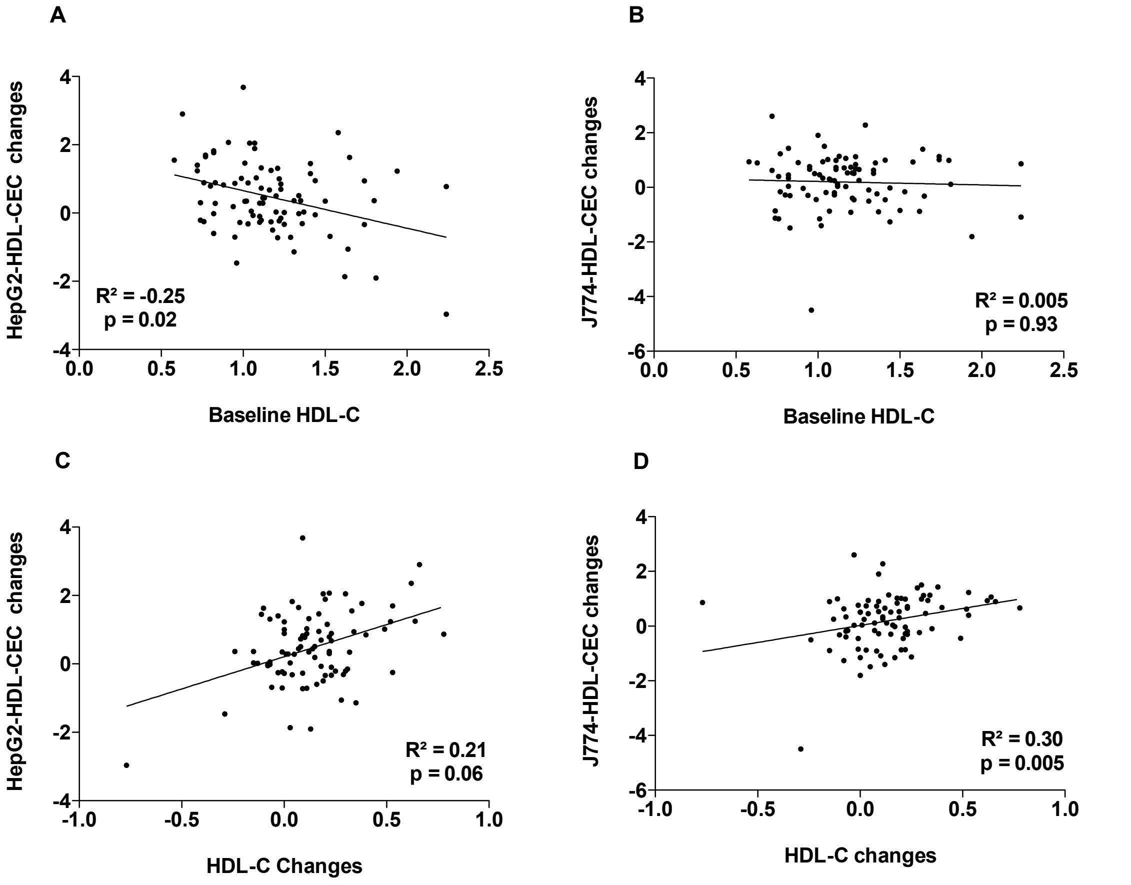

Figure S3. Association between HepG2-HDL-CEC changes and baseline Apolipoprotein A-1 (A) and Apolipoprotein A-1 changes (C) and J774-HDL-CEC changes with baseline Apolipoprotein A-1 (B) and Apolipoprotein A-1 changes (D).

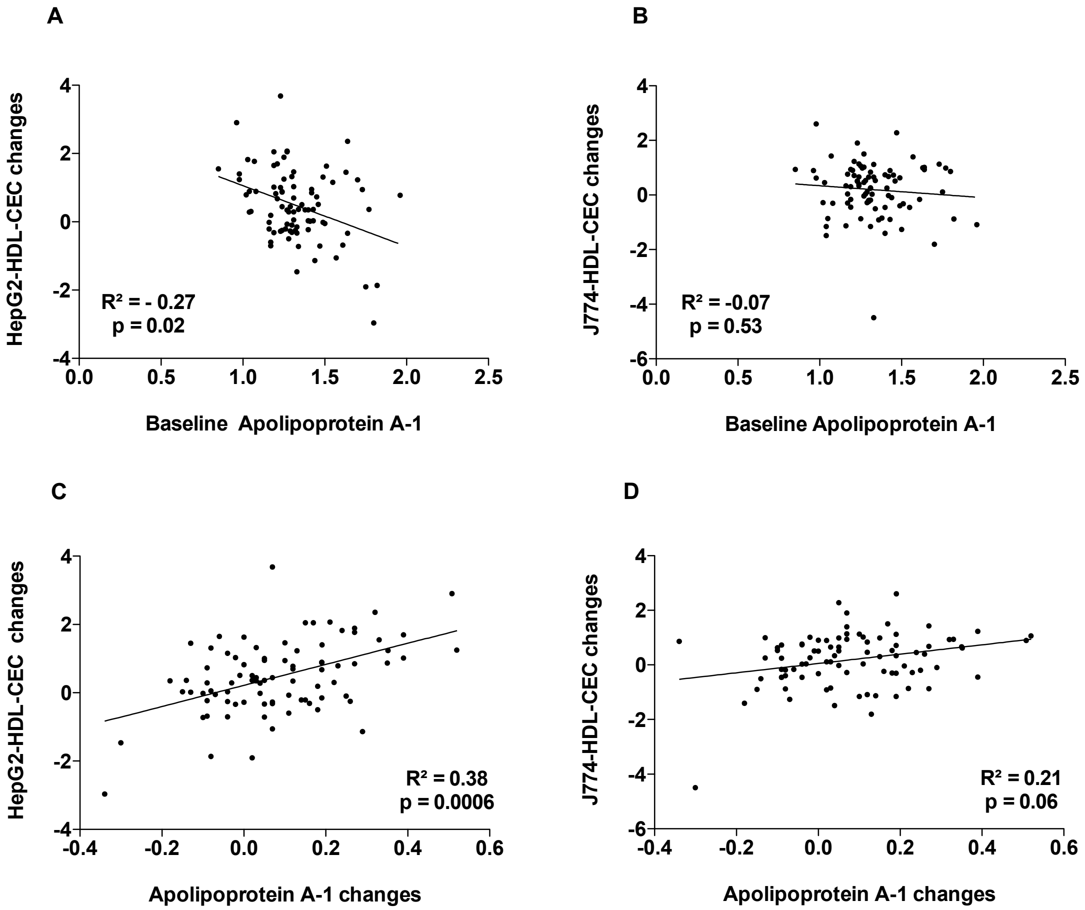

Figure S4. Association between HepG2-HDL-CEC changes and baseline visceral AT (A) and visceral AT changes (C) and J774-HDL-CEC changes with baseline visceral AT (B) and visceral AT changes (D).

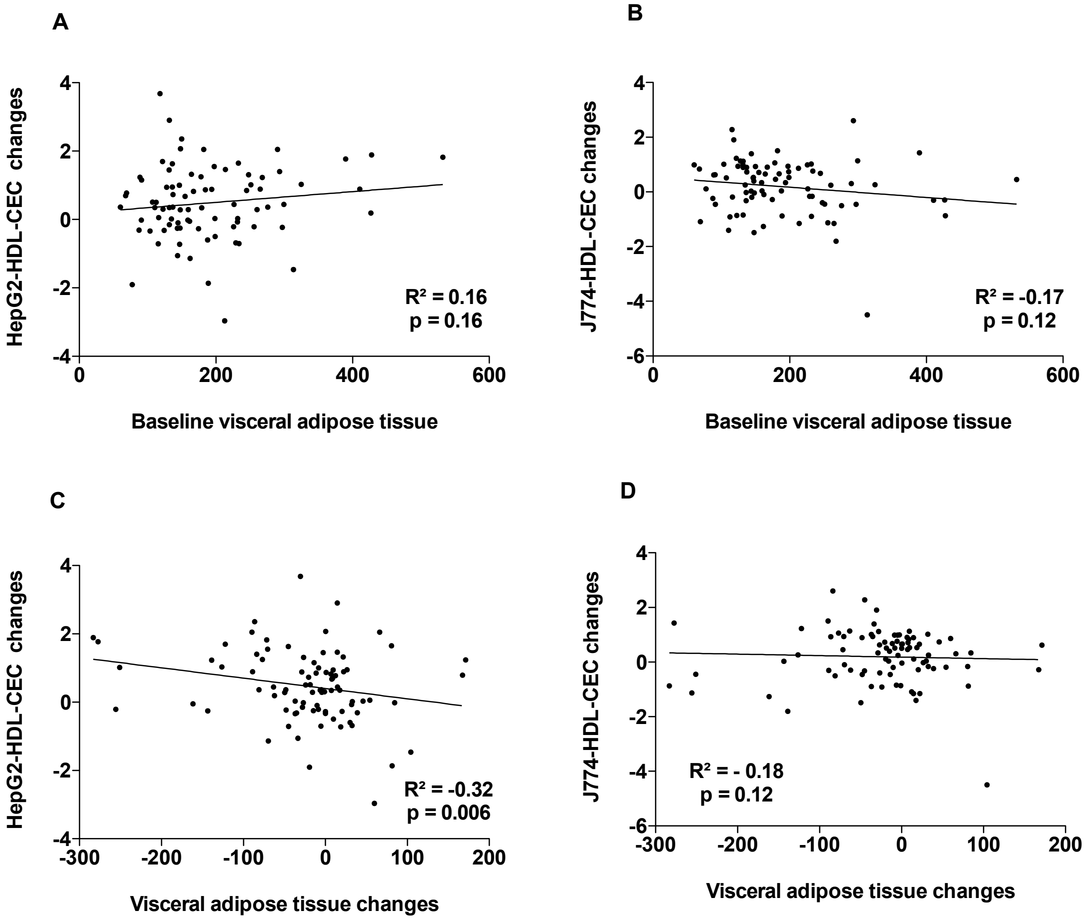

Figure S5. Association between HepG2-HDL-CEC changes and baseline cardiac AT (A) and cardiac AT changes (C) and J774-HDL-CEC changes with baseline cardiac AT (B) and cardiac AT changes (D).

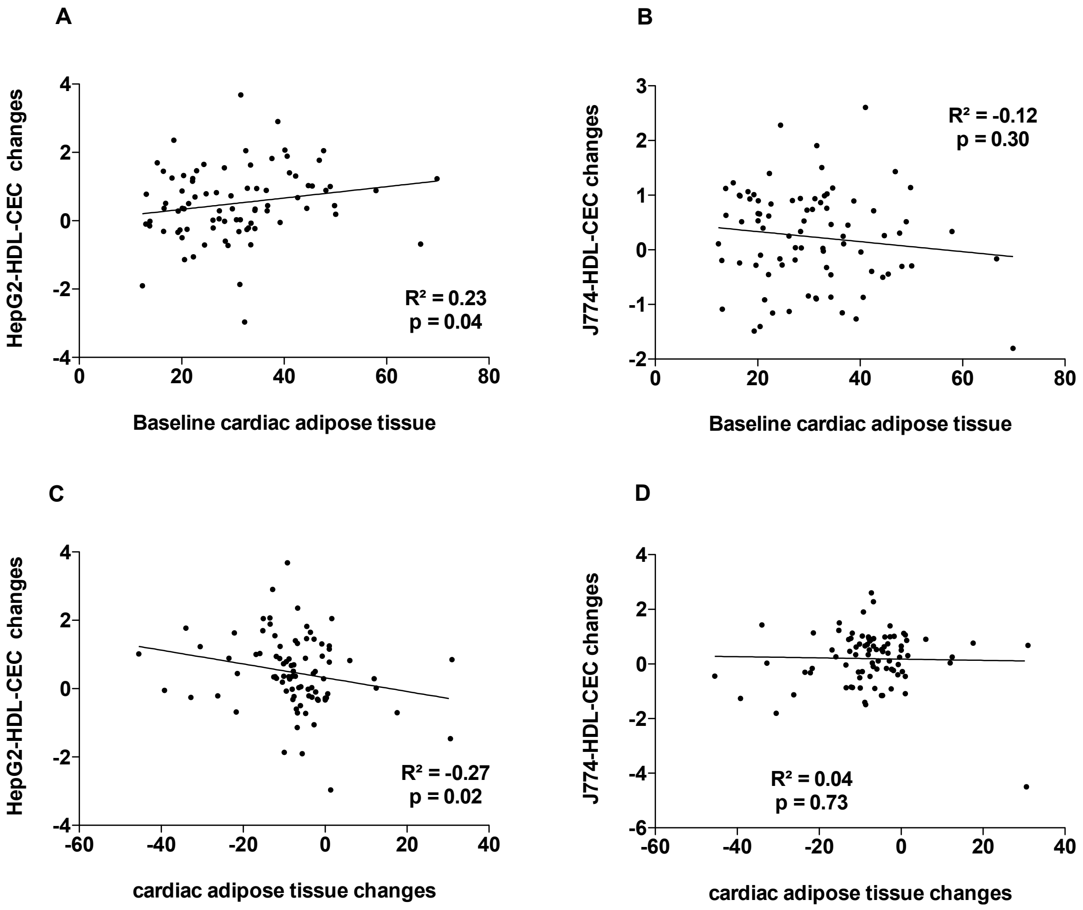

Figure S6. Association between HepG2-HDL-CEC changes and baseline epicardial AT (A) and epicardial AT changes (C) and J774-HDL-CEC changes with baseline epicardial AT (B) and epicardial AT changes (D).

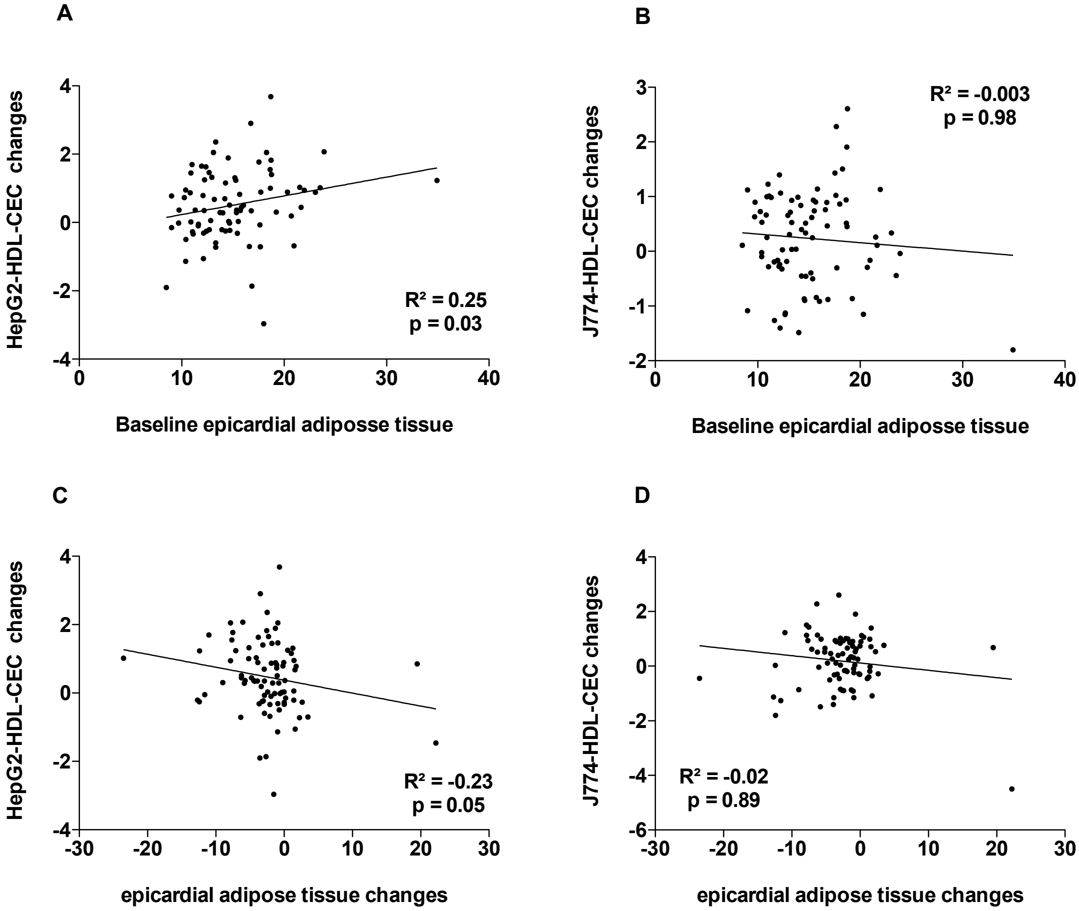

Figure S7. Association between HepG2-HDL-CEC changes and baseline pericardial AT (A) and pericardia AT changes (C) and J774-HDL-CEC changes with baseline pericardia AT (B) and pericardia AT changes (D).

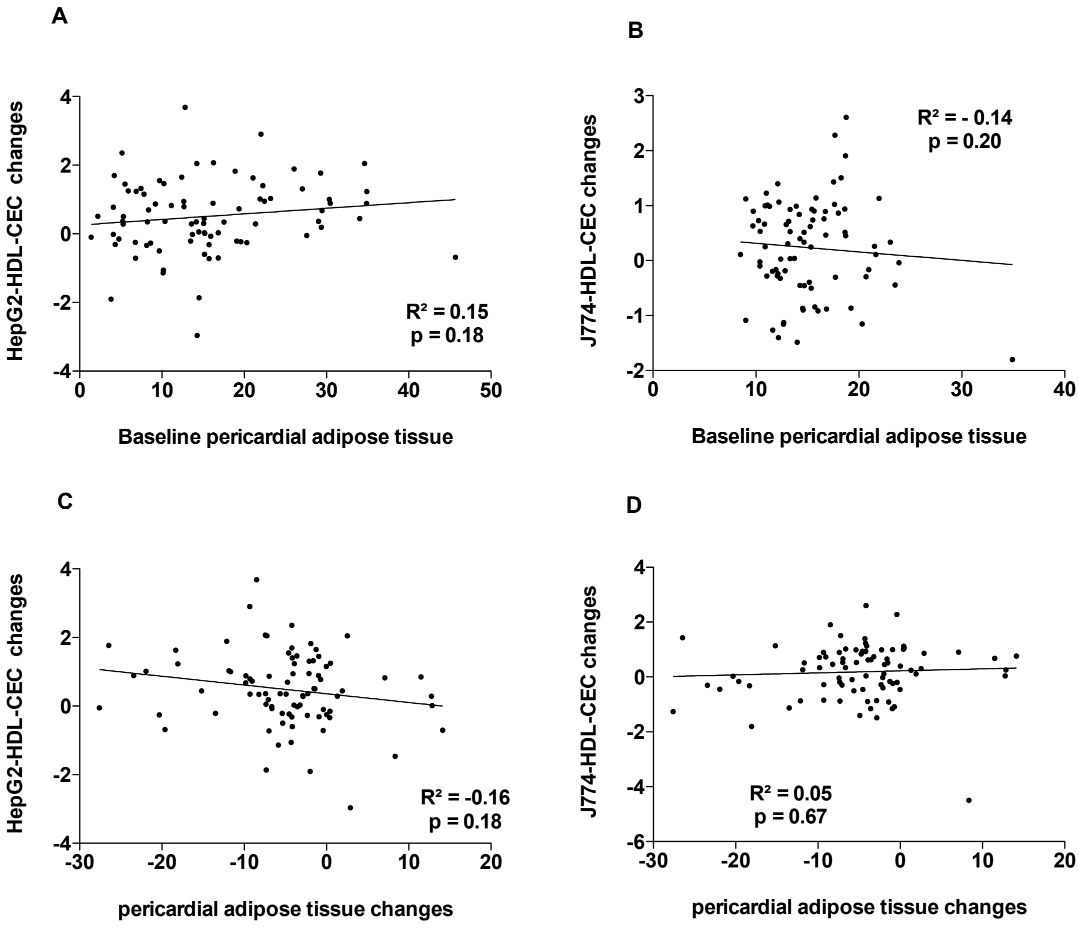

Figure S8. Association between HepG2-HDL-CEC changes and baseline VO_2 peak (A) and VO_2 peak changes (C) and J774-HDL-CEC changes with baseline VO_2 peak (B) and VO_2 peak changes (D).

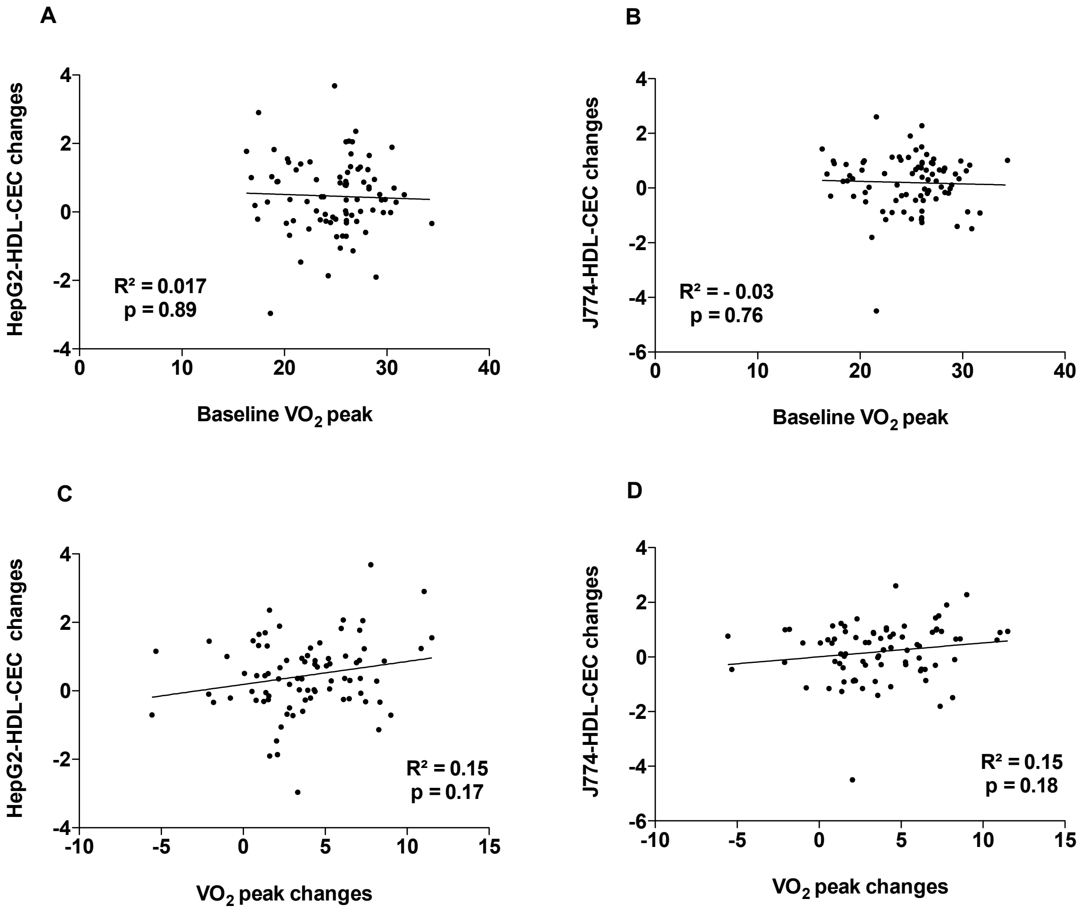

Supplement: Supplementary file 1 — Figure S1. Association between HepG2–high‐density lipoprotein cholesterol efflux capacity (HepG2‐HDL‐CEC) changes and baseline HepG2‐HDL‐CEC (A) and J774‐HDL‐CEC changes with baseline J774‐HDL‐CEC (B). Figure S2. Association between HepG2–high‐density lipoprotein cholesterol efflux capacity (HepG2‐HDL‐CEC) changes and baseline high‐density lipoprotein cholesterol (HDL‐C; A) and HDL‐C changes (C) and J774‐HDL‐CEC changes with baseline HDL‐C (B) and HDL‐C changes (D). Figure S3. Association between HepG2–high‐density lipoprotein cholesterol efflux capacity (HepG2‐HDL‐CEC) changes and baseline apolipoprotein AI (apo AI; A) and apo AI changes (C) and J774‐HDL‐CEC changes with baseline apo AI (B) and apo AI changes (D). Figure S4. Association between HepG2–high‐density lipoprotein cholesterol efflux capacity (HepG2‐HDL‐CEC) changes and baseline visceral adipose tissue (AT; A) and visceral AT changes (C) and J774‐HDL‐CEC changes with baseline visceral AT (B) and visceral AT changes (D). Figure S5. Association between HepG2–high‐density lipoprotein cholesterol efflux capacity (HepG2‐HDL‐CEC) changes and baseline cardiac adipose tissue (AT; A) and cardiac AT changes (C) and J774‐HDL‐CEC changes with baseline cardiac AT (B) and cardiac AT changes (D). Figure S6. Association between HepG2–high‐density lipoprotein cholesterol efflux capacity (HepG2‐HDL‐CEC) changes and baseline epicardial adipose tissue (AT; A) and epicardial AT changes (C) and J774‐HDL‐CEC changes with baseline epicardial AT (B) and epicardial AT changes (D). Figure S7. Association between HepG2–high‐density lipoprotein cholesterol efflux capacity (HepG2‐HDL‐CEC) changes and baseline pericardial adipose tissue (AT; A) and pericardia AT changes (C) and J774‐HDL‐CEC changes with baseline pericardia AT (B) and pericardia AT changes (D). Figure S8. Association between HepG2–high‐density lipoprotein cholesterol efflux capacity (HepG2‐HDL‐CEC) changes and baseline cardiorespiratory fitness (VO2 peak; A) and VO2 pea [file JAH3-7-e008681-s001.pdf]
